# High Spatial Resolution Detector System Based on Reconfigurable Dual-FPGA Approach for Coincidence Measurements

**DOI:** 10.3390/s24165233

**Published:** 2024-08-13

**Authors:** Marco Cautero, Fabio Garzetti, Nicola Lusardi, Rudi Sergo, Luigi Stebel, Andrea Costa, Gabriele Bonanno, Enrico Ronconi, Angelo Geraci, Igor Píš, Elena Magnano, Maddalena Pedio, Giuseppe Cautero

**Affiliations:** 1 Dipartimento di Fisica, Università degli Studi di Trieste, 34127 Trieste, Italy; marco.cautero@units.it; 2 Elettra Sincrotrone Trieste S.C.p.A., S.S. 14-km 163,5 in AREA Science Park, 34149 Basovizza, TS, Italy; rudi.sergo@elettra.eu (R.S.); luigi.stebel@elettra.eu (L.S.); giuseppe.cautero@elettra.eu (G.C.); 3 DEIB (Dipartimento di Elettronica, Informazione e Bioingegneria), Politecnico di Milano, 20133 Milano, Italy; fabio.garzetti@polimi.it (F.G.); andrea1.costa@polimi.it (A.C.); gabriele.bonanno@polimi.it (G.B.); enrico.ronconi@polimi.it (E.R.); angelo.geraci@polimi.it (A.G.); 4 CNR-IOM (Istituto Officina dei Materiali), Strada Statale 14-km 163,5 in AREA, 34149 Basovizza, TS, Italy; pis@iom.cnr.it (I.P.); magnano@iom.cnr.it (E.M.); 5 CNR-IOM (Istituto Officina dei Materiali), Via Pascoli s.n.c., 06123 Perugia, Italy; pedio@iom.cnr.it; 6 INFN (Istituto Nazionale di Fisica Nucleare), Sez. di Perugia, Via Pascoli s.n.c., 06123 Perugia, Italy; 7 INFN (Istituto Nazionale di Fisica Nucleare), Sez. di Trieste, Via Valerio 2, 34127 Trieste, Italy

**Keywords:** time-to-digital converter (TDC), time-mode, cross delay-line (CDL), time-resolved experiments, digitization of sensor data, field-programmable gate array (FPGA)

## Abstract

Time-resolved spectroscopic and electron–ion coincidence techniques are essential to study dynamic processes in materials or chemical compounds. For this type of analysis, it is necessary to have detectors capable of providing, in addition to image-related information, the time of arrival for each individual detected particle (“x, y, time”). The electronics capable of handling such sensors must meet requirements achievable only with time-to-digital converters (TDC) with a resolution on the order of tens of picoseconds and the use of a field-programmable gate array (FPGA) to manage data acquisition and transmission. This study introduces the design and implementation of an innovative TDC based on two FPGAs working symbiotically with different tasks: the first (AMD/Xilinx Artix^®^ 7) directly implements a TDC, aiming for a temporal precision of 12 picoseconds, while the second (Intel Cyclone^®^ 10) manages the acquisition and connectivity with the external world. The TDC has been optimized to operate on eight channels (+ sync) simultaneously but is potentially extendable to a greater number of channels, making it particularly suitable for coincidence measurements where it is necessary to temporally correlate multiple pieces of information from various measurement systems.

## 1. Introduction

Time-resolved spectroscopic and electron–ion coincidence techniques are important for studying dynamic processes in materials or chemical compounds [1,2]. In these experiments, multiple measurement events coming from several sensors are examined, and by analyzing the temporal correlation, various information are extracted. For this type of analysis, it is necessary to have sensors capable of providing, in addition to image-related information, the time of arrival for each individual detected particle (“x, y, time”), and they should be part of a “detection environment” that includes multiple sensors and measurement points.

These sensors, in extreme cases, may require achieving spatial resolutions in the order of tens of microns, time accuracies in the scale of tens of picoseconds, and the detector must be capable of correlating multiple events from other sensors and digital signals for each acquired event [3,4].

One of the most utilized architectures, especially when sensitivity to single particles (e.g., photons, electrons, and ions) and 2D information are required, involves using a charge multiplier, typically a microchannel plate (MCP), followed by a charge pulse detector. This setup provides spatial and temporal information about the particle’s impact point on the MCP [5].

To date, the sensors that best suit this type of detection are based on cross-delay lines (CDL), which utilize the propagation time of the charge pulse produced by MCPs on orthogonal delay lines to derive both spatial information, achieving resolutions in the range of several tens of microns, and temporal information, with precision in a few tens of picoseconds [6].

These types of sensors can achieve desired counting rates, reaching several MCounts/s [7], but only if accompanied by real-time processing electronics that can execute multiple parallel operations simultaneously at a high processing frequency. Consequently, these sensors inevitably depend on the utilization of FPGAs.

Therefore, the most sophisticated acquisition systems involve an FPGA orchestrating data from one or more TDCs connected to CDL sensors or other measurement points, integrating processing intelligence, finding the necessary correlations, and executing a number of digital filters to exclude inconsistent data (also to reduce data throughput for transmission), and transmitting pre-processed coordinates to a CPU.

In this work, we present such a detection system that takes a further significant step forward by leveraging the capabilities of FPGAs not only for their computing power but also for their intrinsic ability to manage and calibrate time delays of a few picoseconds. This approach departs from the usual “application specific integrated circuit (ASIC) T/D converter connected to the FPGA” paradigm, which, while achieving excellent temporal resolution, is strictly tied to the capabilities of the component (ASIC) implementing the TDC. Instead, the proposed architecture integrates the sensor’s time measurement operation within the FPGA itself, not only achieving the timing performance of the best ASIC-based TDCs but also introducing reconfigurability and adaptability to each individual experiment.

In particular, we describe the design and implementation of an innovative time-resolved 3D sensor (x, y, time) based on two FPGAs working symbiotically with different tasks: the first (Artix^®^ 7) directly implements a TDC aiming for a temporal precision of 12 picoseconds, while the second (Cyclone^®^ 10) manages the acquisition and connectivity with the external world, as well as all event detection selection and processing intelligence operations. Thanks to this architecture, it is possible to perform real-time operations on each individual event acquired by the sensor, as well as on the final image.

Furthermore, a system where all acquisition and management operations are delegated to two FPGAs allows for a complete redefinition of each input, adapting to entirely different experiments. This includes scenarios where there can be simultaneous acquisition from multiple CDLs or from N different time of flight sensors or a combination of sensors with signals from photodiodes or light sources machines such as synchrotrons or lasers. Finally, the ongoing research in the field of FPGAs, aimed at handling increasingly faster signals, hints at significant advancements in this approach. For instance, the capability to receive and distinguish multiple events acquired by the same sensor within time intervals shorter than a nanosecond.

In the following sections of this article, we describe, for the first FPGA, how delays between logic gates within the FPGA are utilized to create an advanced delay measurement system and how this low-level operation can be implemented using FPGA programming tools. For the second FPGA, we delineate all the algorithms, executed at a lower level (HDL—hardware description language), which transform the pulses from CDL sensors and other measurement points into images correlated with external events.

Another aspect that is discussed concerns the implementation of a high-speed protocol that enables communication between the two FPGAs. Two different interfaces have been tested and compared in order to minimize the crosstalk of the communication signals on the time measurement cores.

We conclude this article by presenting a series of measurements conducted both in the laboratory and in the field, using synchrotron light in a typical experiment where high temporal resolution is required. The first measurements we show are aimed at demonstrating the excellent results in terms of pure time precision. We then show how this precision, due to the conversion work carried out by the master FPGA, translates into an excellent particle detection system of the “x, y, time” type based on cross-delay anodes, verified both with a specially designed setup and in a vacuum chamber with MCPs equipped with an appropriate mask. The final test shows how the new TDC system, employed in a “photon in/photon out” time-resolved experiment with synchrotron radiation, improves the measurements compared to a TDC based on ASIC [8].

## 2. Materials and Method

As anticipated, in correlation and coincidence measurements, a single sensor is not sufficient; instead, a “sensors system” is required, orchestrated by an intelligence that in more demanding cases, must reside on an FPGA. Since, for these sensors, it is often necessary to have temporal information regarding the time of acquisition, the analog signal processing from the front-end must include elements that preserve temporal information about the time detection. This means that one cannot rely on simple discriminators, which would be strongly influenced by the amplitude of the signals generated by the sensors, but it is necessary to pass—for analog signals—through constant fraction discriminators (CFDs).

Each CFD emits digital pulses related to the arrival time, which are then sent to the FPGA-TDC, which converts them into numerical values representing delays between the various received signals. These numerical values are then sent to the FPGA-Master, which transforms them into images after performing a series of filters and processing steps. The images are then communicated to a PC for further high-level processing.

It is important to consider that the performance metrics of a FPGA-TDC are highly dependent on the routing of signals within the core fabric [9]. Moreover, the strict timing constraints imposed on the TDC-FPGA to reach optimal precision and linearity severely increase the compile time. Consequently, any modification to the design, such as the introduction of the processing, could potentially degrade performance and dramatically slow down the compilation process. However, by separating logically independent tasks—such as time-to-digital conversion and data processing—between two FPGAs, a modular strategy can be employed. This approach mitigates potential issues that may arise when altering the data-processing datapath. Furthermore, this strategy facilitates the replacement of individual components, whether by incorporating a different FPGA or by implementing the TDC on an ASIC.

One of the most important tasks of the FPGA-Master is to systematically eliminate all events that are not experimentally admissible (we see various examples later). Only due to this pre-processing it is possible to perform high-speed acquisitions without being limited by the transfer speed with the PC. However, the data flow between the two FPGAs is not yet filtered, so it is very high, and it was necessary to implement an appropriate communication bus between the two FPGAs. Given the criticality of this element, an entire paragraph is dedicated to this aspect.

Therefore, the roles are well-defined: FPGA-TDC ensures that the signal acquisition times from various sensors are measured with the utmost precision (order of tens of picoseconds), and the FPGA-Master transforms these times into “information” (time of flight, points of an image, acquisition triggers, etc.) by controlling their experimental consistency and performing necessary processing steps that can vary from experiment to experiment. This is one of the most important properties of this type of approach, namely, the ability to completely change processing algorithms based on experimental needs that may change entirely from one experiment to another.

**Figure 1:**
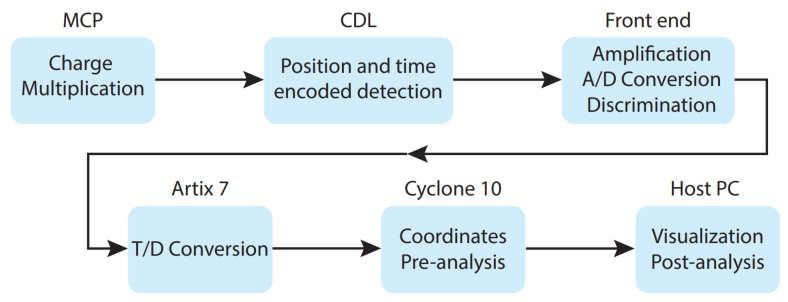
Acquisition chain for single particle detection.

The primary components of the imager are here described in detail and represented in Figure 1. The system is composed of a CDL detector with a MCP used for charge multiplication, an analog-to-digital front-end stage used to convert the analog pulses at the output of the CDL lines into digital pulses, and a fully-digital part consisting of two different FPGAs, a Xilinx (now AMD) 28-nm Artix^®^ 7 used for the TDC implementation (FPGA-TDC) and an Intel 28-nm Cyclone^®^ 10 as control logic and real-time data processing (FPGA-Master). The latter is connected to a personal computer through a 1 Gbit/s Ethernet network. Table 1 reports the resource utilization for the two FPGAs. It is evident that the logic resources offered by these two FPGAs are sufficient to implement additional firmware upgrades specific to the case study. While this justifies the choice of the Cyclone^®^ 10 as the FPGA-Master within a modular instrument, the selection of the Artix^®^ 7 as the TDC is based on previous studies [10].

The radio-frequency (RF) amplifiers and CFDs in the A/D stage preserve the information about the pulses’ arrival times against variations in their amplitude. The FPGA-TDC receives the CFD outputs and calculates the arrival time of the incoming pulses in relation to an external or internal reference signal. In order to make it simple to reconstruct 3D images (x, y, time), the timing information is then transferred to the FPGA-Master, which handles system control and data processing activities targeted at real-time determination of spatial coordinates and correlation to the corresponding timestamps. Additionally, the user datagram protocol (UDP) handshake between the control and data acquisition PC and the FPGA-Master is managed.

An in-depth description of the various components of the imager is provided in the following sections.

### 2.1. Imaging Detector

While the use of the architecture based on two FPGAs here described is potentially applicable in techniques where the sensor is one-dimensional (and cases where this occurs will be mentioned), the more interesting scenario involves the TDC associated with an imaging detector for measurements resolved both in space and time. For this reason, we particularly address this case, and it is important to summarize the operating principles of such a particle detector.

**Figure 2:**
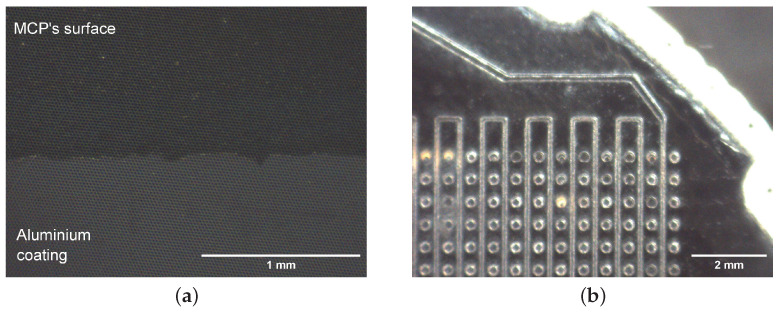
(**a**) Microscope image of the MCPs. The contrast of the image was increased to distinguish the deposition of aluminum in the lower half of the device. (**b**) Microscope image of the cross-delay line detector realized onto a printed circuit board. Contact vias with the orthogonal delay line are visible as small circles.

To detect the signal generated by a single particle while preserving both spatial and temporal information, a F1217-011 MCP (Hamamatsu Photonics, Shizuoka Prefecture, Japan) is used. A bidimensional 280 nm LED array illuminates the entire area of the MCP homogeneously. Subsequently, to measure the spatial resolution of the detector, a patterned aluminum mask, visible in the lower half of Figure 2a, is deposited via chemical vapor deposition directly onto the MCP’s surface.

**Figure 3:**
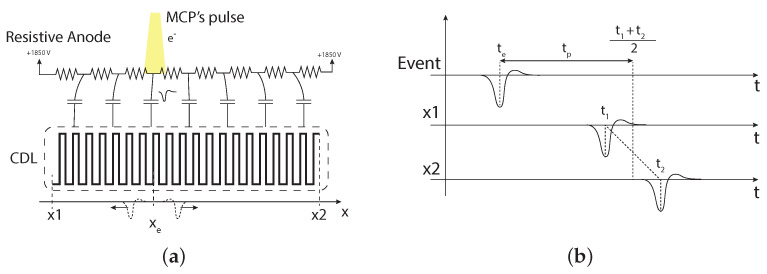
(**a**) Detection of an MCP pulse on a single delay-line. (**b**) Timing of the events generated by the EM pulse.

Referring to the drawing shown in Figure 3, it is evident how temporal information about the arrival of pulses provides spatial information. The cloud of electrons generated by the MCP at the point of arrival translates into an electromagnetic pulse that propagates in two directions along two orthogonal, electrically isolated transmission lines.

Focusing on one line for simplicity, shown in Figure 3b, the temporal and spatial coordinates (still expressed in temporal units) are determined by the arrival times of the EM pulse at the two ends of the propagation line, as follows:

(1) $$x_{e}=t_{1}-t_{2},$$

(2) $$t_{e}=\frac{t_{1}+t_{2}}{2}-t_{p}$$

where $t_{1}$ and $t_{2}$ are the measured arrival times at the first and second terminal, respectively, and $t_{p}$ is the total propagation time of the pulse along the delay line (for our purposes, these can be expressed later on in terms of bins of the TDC without loss of generality). According to this equation, if $t_{1}$ is smaller than $t_{2}$ (i.e., $t_{1}$ is measured first), *x* assumes a negative value that corresponds to an event spatially closer to the first terminal. Conversely, if *x* is positive (i.e., $t_{2}$ < $t_{1}$), the event occurred closer to the second terminal. It is important to note that not all values of $t_{1}$ and $t_{2}$ are acceptable. Specifically, for an event to be physically sensible, the following condition must be met:

(3) $$|t_{1}-t_{2}|<t_{p}$$

According to this inequality, the values of *x* must lie between $-t_{p}$ and $t_{p}$, giving a temporal validity window of $2\cdot t_{p}$. To obtain the coordinates in spatial units, the conversion factor *k* is given by

(4) $$k=\frac{l}{2\cdot t_{p}}$$

where *l* is the length of the side of the propagation line.

The same computations are performed on the orthogonal trace, and by combining the two pieces of information, two-dimensional information is obtained. The information becomes 3D when considering that the temporal information about the particle’s arrival concerning any external event is inherently contained in this position measurement.

Furthermore, in this case, it is necessary to define a boundary condition to discard combinations that could not have physically originated from a single event. In particular, this time the condition is the following:

(5) $$t_{xy,min}<t_{x}-t_{y}<t_{xy,max}$$

where $t_{x}$ and $t_{y}$ are the propagation times of the two orthogonal delay lines calculated previously, and $t_{xy,max}$ and $t_{xy,min}$ are the maximum and minimum time intervals that can occur between $t_{x}$ and $t_{y}$ to be considered valid. These parameters depend mainly on the detector’s geometry and material.

### 2.2. Front End

A suitable conditioning stage is required due to the small amplitude of the electromagnetic pulses generated by the CDL, which typically is a few millivolts.

Given that the meander lines function as transmission lines with a typical resistance of 50 Ω, RF amplifiers with matched inputs are the optimal choice for signal amplification. In-house built RF amplifiers are positioned close to each extremity of the two anode lines of the CDL detector to minimize the likelihood of interference from external noise sources. The pulses exhibit a Gaussian shape, with a full width at half maximum (FWHM) of 4 ns and rise and fall times of 2.8 ns, indicating that the signal energy is confined to a bandwidth below 150 MHz. Based on these specifications, each amplifier consists of a cascade of two GALI-S66+ Monolithic Microwave Integrated Circuits (Mini-Circuits, Brooklyn, NY, USA). The overall gain is approximately 43 dB, with cut-off frequencies at −3 dB ranging from 100 kHz to 1 GHz and a flatness of ±0.5 dB from 1 MHz to 400 MHz.

The output of each RF amplifier is connected to the input of a custom developed CFD, which converts the amplified analog pulses—now with amplitudes ranging between 0.5 and 2 V—into digital signals compatible with the low-voltage differential signaling (LVDS) logical input standard of the TDC. This conversion is achieved by extracting the time position of the peak of the Gaussian input signal, regardless of amplitude variations. The pulses generated by the MCPs mounted above the two orthogonal anodes in the CDL detector exhibit varying amplitudes, depending on the MCPs’ operating voltage and the event count rate. Consequently, straightforward approaches like standard threshold discriminators, which offer sufficient walk error rejection, are not suitable for accurately converting an event’s arrival time information into a digital format [11]. A comprehensive characterization of this front-end has been conducted in previous studies [12].

### 2.3. FPGA-TDC

A tapped delay-line-based TDC (TDL-TDC) [10] provides timestamps with resolution (LSB) of 2.34 ps over a full scale-range (FSR) of 0.145 ms, single-shot channel precision below 12 ps r.m.s., minimum dead-time of 7 ns, and an integral non-linearity error (INL) lower than 4 ps over a dynamic range of 500 ns. These features can perform as well as systems that are currently at the cutting edge when it comes to final image reconstruction. Table 2 provides a summary of the implemented TDC performance.

The time is measured by counting how many bins the signal passes through between the “start” and “stop” of the measure. In theory, the TDL-TDC is made up of a chain of buffers (bins or taps) that are employed as delay cells in a delay line. The TDL-TDC essentially consists of a chain of buffers with flip-flops acting as output registers. The TDL, in this sense, turns the interval restricted by the two time markers “start” and “stop” into a number. The “start” event, which is simply a binary transition, is propagated along the buffer sequence, whose outputs are the inputs of the flip-flops. The line where the “stop” marker, ending the interval, occurs is the flip-flop clock gate (CLK). The flip-flop chain simultaneously records the output status of every buffer on the “stop” signal, returning as output (Q) a sequence of ‘1’s with a length corresponding to the length of the time interval being measured. Finally, the measurement value’s thermometric-code format is decoded into binary form.

**Figure 4:**
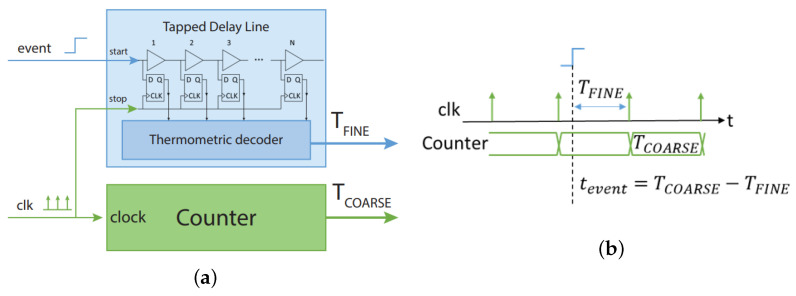
(**a**) Detail of TDC internal logic for a single channel. Timestamps are obtained from a fine measurement of a TDL and coarse measurement of a counter. (**b**) Principle of the Nutt-Interpolation on a TDL-TDC.

The propagation delay of the buffers making up the delay line determines the resolution of TDC in this design. Accordingly, the performance of this fundamental architecture would be unacceptably poor due to two factors: first, it is constrained by the propagation delays of the FPGA device’s bins (tens of picoseconds for the 28-nm), and second, by the variations in the propagation times through the various buffers (up to three or four times the mean propagation delay), which are also caused by the layout of those buffers [13]. These inescapable structural issues of the device have an adverse effect on resolution and linearity, which calls for the use of specialized sub-interpolation and calibration procedures [14]. More specifically, the calibration enables the device to regain outstanding linearity while the sub-interpolation makes up for the lack of native resolution (reaching an LSB of units of picoseconds for the 28-nm). The installed TDL-TDC may additionally achieve a dynamic range extended to over ten seconds due to the Nutt-Interpolation approach [15], which works in concert with a digital counter to increase the FSR. The “stop” of the TDL-TDC is connected to a clock that likewise powers the digital counter, as seen in Figure 4a. By combining a fine measurement ($T_{FINE}$), made by the TDL-TDC, which can be viewed as the time interval between the instant itself and the clock, and a coarse measurement ($T_{FINE}$), provided by the counter as the counter value itself, it is possible to visualize how the measurement of the instant of occurrence of an event (“start” of the TDL-TDC in Figure 4b) can be understood.

Depending on the requirements, the FPGA-TDC can be programmed with firmware featuring different number of channels. In this case, eight channels are provided. Each channel is called “STOP” (the capitalization is used to distinguish from the “stop” signal of the TDL-TDC). Four “STOP” channels are typically used to connect with the four “stop” signals, one for each end of the two meanders of the CDL. The remaining “STOP” channels may be connected to any other signal to provide extra information or time reference.

The TDC has a pipeline design that enables it to reach a 7 ns dead-time. Due to the minimal dead-time, the system is more effective overall when numerous successive events arrive on the same input because all of the CDL’s pulses may be collected without loss.

A AMD Artix^®^ 7 XC7A200T-1FBG484 FPGA is used to implement the TDC firmware, which is intended to ensure excellent signal integrity while minimizing ground-bounce, channel crosstalks, and clock jitters. The excellent quality of the image and the complete absence of local distortions are primarily reflected in the high performance achieved in terms of precision (12 ps r.m.s.) and integral non-linearity (4 ps over a dynamic range of 500 ns) [12].

### 2.4. FPGA-Master

In addition to managing the communication with the FPGA-TDC on one side and with the acquisition environment on the other, which is discussed in the next paragraph, the FPGA-Master, an Intel Cyclone^®^ 10CX105YF780I6G, also plays a role in the pre-processing of the data received from the FPGA-TDC.

In fact, in addition to performing the operations that transform the flow of millions of events per second from the FPGA-TDC into image points, each of which is temporally correlated with other signals, the FPGA-Master must perform a control on the events detected on each channel, based on the relative delays between their arrival times, in order to discard events that are considered “not valid”. These controls are described in the Section 2.1 relative to the CDL.

Depending on the type of experiment, this initial screening serves different purposes. In experiments with low count rates—although still high enough to be unfeasible for real-time processing on a PC—such as coincidence measurements and time-resolved X-ray absorption spectroscopy (TR-XAS), time-correlated image points are crucial for identifying adequate regions of interest. Conversely, in experiments involving imaging techniques where count rates reach the order of millions per second, an algorithm to filter out invalid signals is essential to prevent the transmission of excessive data, thereby avoiding the risk of saturating the Ethernet connection.

In certain scenarios, particularly for coincidence measurements where not all signal correlations are known a priori, a more in-depth analysis of the collected data is required. To this end, a copy of the raw timestamps is sent directly to the PC via an optional secondary Gigabit Ethernet connection prior to on-board processing, which still provides useful real-time information regarding the live evolution of time-resolved phenomena.

To ensure that processing remains independent from the incoming data format while still leveraging the modular architecture, all processing within the FPGA-Master utilizes a custom timestamp format. Consequently, the timestamps of signals received from the FPGA-TDC are re-binned and formatted into an extended 56-bit format using an additional counter. This approach standardizes the timestamp data, facilitating consistent processing regardless of the original data format.

In addition to the more complex correlation-oriented features, which are discussed later, the FPGA-Master is equipped with basic functions that enable the system to adapt each input to the specific scientific case under consideration. Specifically, the initial processing of the timestamps includes a dead-time module and an offset compensation module. These two modules operate independently on each channel and are designed to eliminate spurious events caused by improper connections between the experimental setup and the electronics. Examples of such issues include reflections resulting from impedance mismatches and incorrect correlations due to significant differences in cable lengths.

Following these two modules, the FPGA-Master performs the actual processing for the correlation measurements. As described by the conditions in Equations (3) and (5), the main discriminator for these operations is the temporal distance between the events under consideration. For this reason, the FPGA-Master takes two timestamps coming from two distinct channels and checks whether their difference resides within a specified interval. If this condition is satisfied, the two timestamps are considered to be correlated and are used to compute the coordinates. Otherwise, the oldest between the two timestamps is discarded.

Finally, after the temporal and spatial coordinates are computed, the FPGA-Master allows us to use any of the input channels as the START input mentioned before. For this processing, the last measured timestamp of the reference channel is registered and subtracted from the other channels. If no channel is chosen as a reference, all the timestamps are referred to the beginning of the acquisition.

### 2.5. Communication between FPGA-Master and FPGA-TDC

Crosstalk between connections can negatively impact the precision of the measured time intervals in a TDC implemented within an FPGA. This issue is pertinent both at the printed circuit board (PCB) level and, more subtly, within the core fabric of the FPGA itself. To mitigate these effects, careful planning and routing are essential at every stage of the design process. In addition to the input channels and clock signals necessary for the proper functioning of the TDC logic, the main communication interface between the two FPGAs also plays a crucial role in managing and reducing interferences. For this reason, two different interfaces have been implemented and compared. The first interface is based on a parallel pseudo AXI-stream bus, while the second leverages the transceivers embedded in both FPGAs.

**Figure 5:**
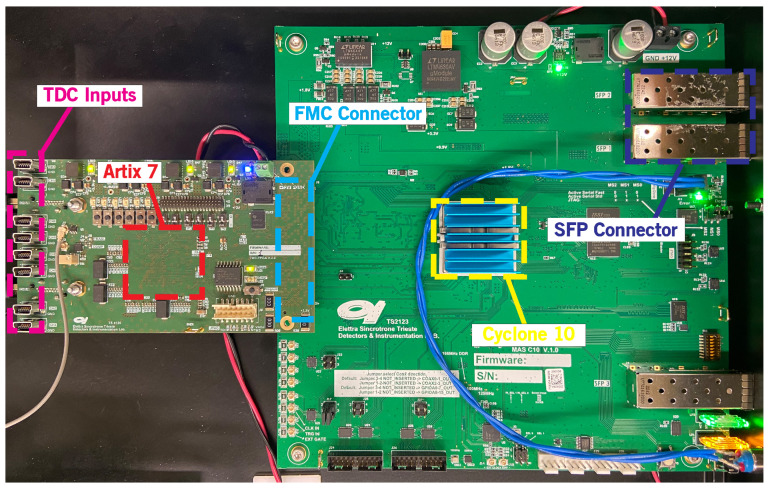
PCBs hosting the FPGA-TDC and FPGA-Master connected using a FMC Connector.

Signal integrity at the PCB level is achieved using impedance matched traces and an FPGA mezzanine card (FMC) connector, as shown in Figure 5. This connector is equipped with both LVDS pairs for parallel communication and high-speed serial lanes for transceiver-based protocols.

Timestamps generated by the TDC FPGA are inserted in chronological order into a 32-bit wide parallel bus. The time interval measurements are encoded using 26 bits, while the remaining 6 bits serve as a header, indicating the channel that registered the event and whether the timestamp represents a fine or coarse measurement. A thorough description of this structure can be found in [16].

For the first communication interface—i.e., the modified AXI-stream bus—32 differential pairs replicate the information of the parallel bus, while two additional pairs are used as data_valid and data_ready flags. The maximum raw throughput in terms of timestamps/s is determined by the clock’s frequency, which, in this case, was set to 100 MHz. The second interface leverages Intel’s and AMD’s IP-Cores for high-speed serial protocols [17]. In particular, the standard used is the gigabit media independent interface (GMII), where data transmission is clocked at 125 MHz with 8-bit data paths for transmission and reception. Since the events are encoded using 32-bit words, the resulting throughput for each transceiver is 31.25 MTimestamps/s.

Although the throughput for the second interface is lower, the effective throughput is constrained by the gigabit connection between the host PC and the Cyclone^®^ 10. In practice, with the extended timestamps of 56 bits and an 8-bit header, this interface can handle a few million counts per second. This throughput is sufficient for the vast majority of experiments, as the stochastic nature of the measured events and the pre-processing performed by the FPGA-Master typically prevent such high count rates from being reached.

## 3. Measurements and Experimental Validation

To test the performance of the newly developed system, several characteristic tests were conducted. The first of these concerns the temporal resolution of the instrument and consequently the spatial resolution of the cross-delay line detector. This measurement was carried out both on the bench, to characterize the instrumentation without any distortions introduced by the specific experimental setup, and in the field, under vacuum conditions, passing through the multiplication of the MCP.

The second test was conducted to compare the system based on FPGA-TDC with a system that uses a commercial ASIC (TDC-GPX, ACAM, Stutensee-Blankenloch, Germany) [8] instead of the FPGA, in the case of a time-resolved pump and probe experiment performed at the BACH beamline of IOM-CNR at the Elettra synchrotron facility (Trieste, Italy) [18,19].

### 3.1. Instrument Characterization

**Figure 6:**
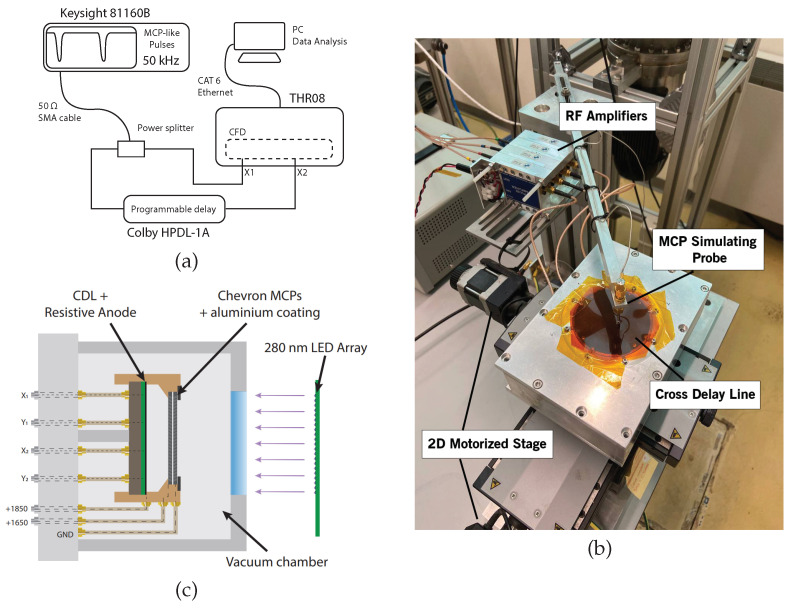
(**a**) Setup used to measure the precision of the TDC and the influence of the communication interface on such measurements. (**b**) Motorized stage with micrometric resolution used for the CDL calibration. (**c**) Drawing of the vacuum chamber used to measure the spatial resolution with the masked MCP.

Tests on the influence of the communication interface on the TDC have been conducted using the setup depicted in Figure 6a. A pulse signal is fed to two channels of the electronics at a time by means of a power splitter to remove the additive jitter introduced by the waveform generator (81160A, Keysight, Santa Rosa, CA, USA). The cumulative distribution of the time intervals measured between the two pulses is used for the evaluation of the accuracy (mean value) and precision (standard deviation) of the TDC. These measurements are repeated for different time intervals due to a programmable delay line (HPDL-1A, Colby Instruments, Bellevue, WA, USA) inserted before one of the channels. Moreover, for this instrument, the temporal jitter was negligible due to the passive nature of the delays.

The second set of measurements aims to evaluate the temporal and spatial resolution of the entire imaging system. To isolate the response of the electronics, a probe capacitively coupled with the CDL was used to simulate the typical impulse from an MCP. The setup is shown in Figure 6b. For this ideal case where all the events occur at an arbitrary point and the signal is not affected by noise (resulting in a certain correlation at the FPGA level), the temporal resolution is equal to the FWHM of the distribution of the arrival times of the EM pulses at the two ends of one delay line. By multiplying this value by the conversion factor *k* from Equation (4), the ideal spatial resolution is obtained.

Due to practical limitations at the borders of the CDL, *k* is computed using a linear regression over a series of known movements: using the same setup described earlier, the probe position is translated by a known distance with the help of a bidimensional motorized stage with micrometric precision mounted underneath the CDL. The slope of the fit is then used as a conversion factor from temporal measurements and spatial ones.

Finally, to integrate the MCP contribution into the resolution measurement, a vacuum chamber is mounted, with supports holding the MCP with the aluminum-deposited mask and a CDL. These two elements are capacitively coupled by means of a resistive anode placed between them, in contact with the CDL. A section of the vacuum chamber is show in Figure 6c.

In this case, the mask deposited onto the MPC’s surface was used to evaluate the spatial resolution of the system: the data near a straight sharp edge of this mask are integrated along a line—perpendicular to the edge itself—with a width of 200 pixels. This results in a line profile that represents the edge spread function (ESF) of the instrument. Considering the Gaussian nature of the TDC’s measurement in this kind of imaging apparatus, the ESF can be modeled by the following formula:

(6) $$\mathrm{ESF}\left(x\right)≃a+b\cdot \mathrm{erf}\left(\frac{x-c}{d}\right)$$

where erf(x) is the error function and *a*, *b*, *c*, and *d* are free parameters [20,21]. In particular, this last term is equivalent to the standard deviation σ of the Gaussian of the system multiplied by a factor $\sqrt{2}$. Such a function is used to fit the line profile, and the *d* parameter is used to evaluate the spatial resolution of the system in terms of its FWHM in μm:

(7) $$\mathrm{FWHM}=2k\sqrt{\ln2}\cdot d$$

where *k* is a conversion factor from pixels to μm, seen earlier.

### 3.2. On-Site Testing

The measurements conducted to demonstrate the improvements introduced by the new electronics, compared to the version using commercial ASIC TDC, involved a laser pump and X-ray probe spectroscopy. Due to the capabilities introduced by the FPGA-based electronics, these measurements can be performed using the synchrotron light pulses from the Elettra synchrotron.

Without going into the details of the experiment, which can be found in [22], it is sufficient to briefly recall how this type of measurement proceeds and how the new electronics are essential: this technique involves studying the dynamic evolution of the physical and chemical properties of materials under non-equilibrium conditions using an intense ultrafast laser pulse, known as a “pump”, which excites the system under study. This is followed, after an adjustable time delay, by “probe” pulses to monitor transient states of the system.

By varying the delay time between the pump and probe pulses, researchers can capture a series of snapshots at different stages of the reaction or process, with time resolution limited by the temporal width of the probe pulses. In the case of 3rd generation synchrotrons such as Elettra, this width is on the order of tens of ps, but the new machines under construction [23] will provide pulses of a few ps, making this technique extremely time-resolved, even with synchrotron light.

In the case of TR-XAS at the BACH beamline, a custom setup was prepared for a specific type of pump–probe measurement [22]. The probe pulses came from the synchrotron facility with a periodicity of ∼2 ns between pulses, while the pump signal was a femtosecond-laser with a variable repetition rate (10 kHz–83.3 MHz) synchronized to the ∼500 MHz ring clock of the facility, i.e., with the electron bunches inside the storage ring synchrotron.

Corresponding to each pump pulse, an X-ray fluorescence emission signal is collected from the samples probed by X-ray pulses. This approach fully exploits the available X-ray photon flux at an approximately 500 MHz repetition rate as a stroboscopic probing sequence, thus providing continuous snapshots of the transient state spectra. The photons are detected using a F4655-13 Fast MCP (Hamamatsu Photonics, Shizuoka Prefecture, Japan). The arrival time of all photons is histogrammed by the acquisition software. By comparing the histograms collected in the absence and presence of the pump signal, and by inserting delays between the pump and probe pulses, the return-to-equilibrium dynamics are determined with time resolution limited only by the width of the probe pulses.

The FWHM width of the measured X-ray pulses corresponds to the length of the storage ring electron bunches, convoluted with the intrinsic uncertainty coming from the measurement of the arrival time.

## 4. Results

Following the characterization tests described in the previous section, the following paragraphs report the results regarding the system’s precision depending on the communication interface, its temporal and spatial resolution under different conditions, and a comparison with electronics based on a commercial ASIC.

### 4.1. Communication Interfaces

**Figure 7:**
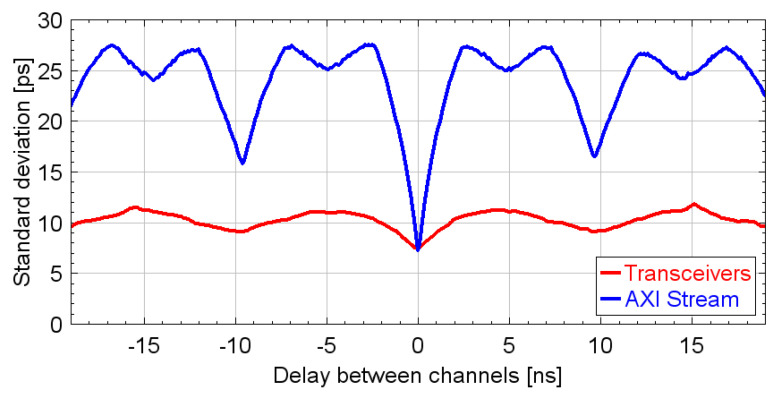
Precision of the FPGA-TDC measured using different communication interfaces with the FPGA-Master.

The temporal precision of the TDC was measured using both the AXI-bus and the transceiver-based communication interfaces, as described in the Measurement section. A comparison between the measurements is shown in Figure 7. As illustrated, the transceiver-based interface significantly improves precision. Specifically, the mean standard deviation measured over a delay span of ±19 ns with this interface is slightly higher than 10 ps, whereas the one measured with the parallel bus interface exceeds 20 ps. Moreover, the precision of the parallel bus interface exhibits periodic peaks with a period of 5 ns. These peaks originate from crosstalk between the 100 MHz clock of the interface and the TDL lines inside the FPGA-TDC. Although different routing could potentially reduce interference, this aspect was not further investigated as it is outside the scope of this work. Instead, by taking advantage of the high-speed communication blocks already embedded in the FPGA’s core fabric, the clock interference was drastically reduced, optimizing the throughput at the same time.

### 4.2. Temporal and Spatial Resolution

**Figure 8:**
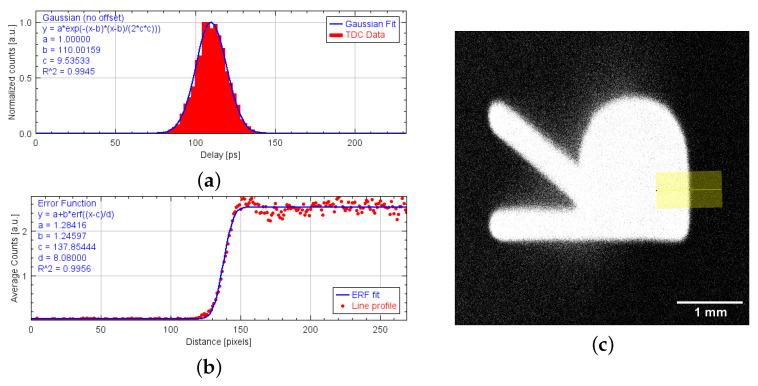
(**a**) Histogram and Gaussian fit of the timestamps for a fixed delay measured with the TDC and probe coupled capacitively with the CDL. (**b**) Line profile of an edge of the image and relative fit with error function. (**c**) Detail of the masked MCP obtained using the imaging setup.

For the evaluation of the system’s temporal and spatial resolution, a bin of 2.344 ps was used and the value of the conversion factor *k*, estimated using a linear regression, is 0.9938 ± 0.0023 [µm/ps]. Figure 8a shows the distribution and Gaussian fit of the timestamps acquired with the electronics and the CDL alone. The fitted Gaussian measures a FWHM of 22.45 ps. Hence, the spatial resolution using this setup is 22.31 µm.

As mentioned, this setup is used to evaluate the spatial resolution of the electronics and the CDL, simulating the electromagnetic cloud generated by an MCP without a vacuum apparatus. Of course, such a measurement presents several differences with an imaging apparatus, and the actual response of an MCP is needed to evaluate the proper spatial resolution of the system.

In Figure 8b,c, the reconstructed image of the masked MCP and the integral profile along the yellow line are shown. According to Equation (7), the fit of the error function yields a *d* parameter of 8.08 pixels. When converted using the appropriate conversion factor and bin size, this value corresponds to a spatial resolution of 31.54 µm.

### 4.3. On-Site Testing

A comparison with an instrument based on a commercial TDC was conducted during a pump–probe experiment at the BACH beamline. The main figures of merit investigated included temporal resolution, accuracy, and the instrument’s capability to enhance statistics by reducing dead times. All of these aspects are crucial in pump–probe experiments. For these measurements, the timestamps of the newly developed electronics used a bin of 9.375 ps, while the previous instrument, based on a TDC based on ASIC, is provided with a fixed bin of 27 ps.

**Figure 9:**
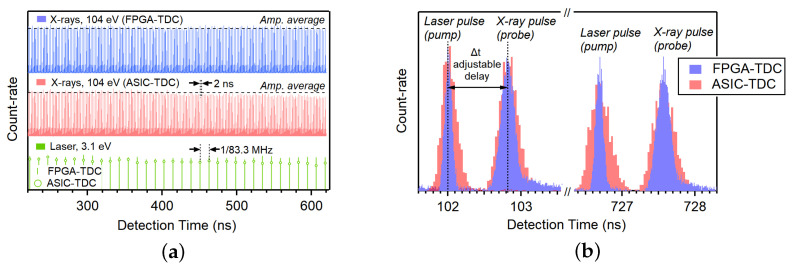
(**a**) Comparison of the time-resolved fluorescence yield from a p-doped silicon sample acquired with TDC implemented on FPGA (blue) and on ASIC (red). (**b**) Enlarged sections of the data shown in (**a**).

Figure 9a,b report the enlarged sections of the histograms recorded over a period corresponding to a complete electron revolution around the synchrotron storage ring (∼864 ns). The results show increased stability of the temporal resolution over long periods for the TDC on FPGA compared to the previous instrument. The temporal resolution of the TDC on ASIC deteriorates over time, resulting in lower and wider peaks with the FWHM gradually increasing from 230 ps to above 330 ps. The temporal resolution of the new electronics reaches 112 ps FWHM, representing an improvement by a factor of 2. As previously reported, the FWHM of the electronics alone remains well below 30 ps. This indicates that the primary contribution to the temporal resolution is from other elements of the apparatus itself, such as the temporal width of the synchrotron bunches or the synchronization electronics present at the beamline.

**Figure 10:**
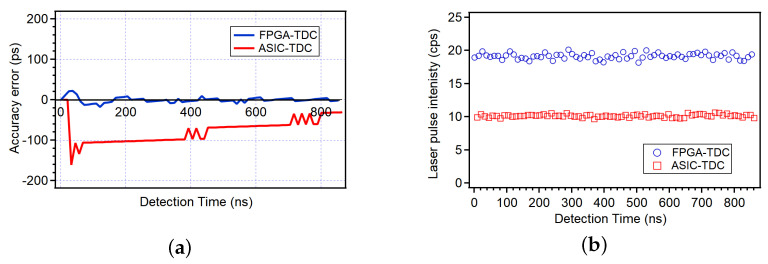
(**a**) Comparison of the accuracy of the TDC implemented on FPGA (blue) and on ASIC (red). (**b**) Comparison of the integrated intensities of laser pulses in counts per second measured under the same experimental conditions.

Major improvements have been achieved in the accuracy as well. Figure 10a shows the accuracy of the two instruments over a complete synchrotron revolution period. The low INL error of the FPGA-TDC over extended time periods is indicative of excellent accuracy, which allows for enhanced pump–probe temporal synchronization.

Finally, the TDC implemented on FPGA allows continuous event registration without significant dead times, aside from the necessary delays to avoid reflections and the pulse pair resolution of the instrument itself. As depicted in Figure 10b, this capability doubles the number of counts per acquisition window. For the purposes of the experiment, this aspect is fundamental as it enhances the measurement statistics and reduces background noise.

## 5. Discussions and State-of-the-Art Comparison

A thorough assessment of the performance characteristics of the primary MCP-based time-resolved detectors with various readouts has been exhaustively presented in [5]. For these detectors, centroid-based techniques are usually employed to compute the particle’s impact position, resulting in a lower bound of the spatial resolution determined by the channel pitch of the MCP. Among these, CDL-detector-based systems are shown to provide the best balance between spatial resolution (up to 20 µm FWHM) and temporal precision (10–100 ps r.m.s.) [24], values that are comparable to those presented in this work.

CDL detectors heavily rely on the TDC’s used performance for both time and spatial resolution. In actuality, the TDC serves as a link between the physically observable phenomena and digital processing; as a result, the primary global system performance metrics, such as resolution, dynamic-range, dead-time, and count rate, are closely correlated with the TDC design in use. In order to extract the timestamps without affecting the performance of the time-interval meter, high-resolution detectors are interfaced to an equally high-resolution time-interval meter (for example, TDC).

Other techniques capable of exploiting event centroiding based on charge division encoding offer a suitable alternative when time measurement is required. Such techniques include multi-anode or pixelated readouts. For the first one, cross strip (XS) anodes [25,26] are among the most recent detection techniques for spatially resolved single-particle detectors. With an additional TDC, temporal information can be measured (still using an additional TDC) either at the anode itself or at the MCP’s electrode, with the latter providing resolution comparable to the CDL approach. Pixelated readouts, on the other hand, enable simultaneous measurement of multiple events, drastically increasing the global count rate. Furthermore, recent developments in this technology allow the achievement of temporal precision just below 200 ps r.m.s. for each pixel [27]. Although comparable with CDL detectors in terms of resolution, both of these approaches are limited to a specific set of measurements. In contrast, a system that relies on time encoding (TDC) can adapt to different experiments, ranging from pump–probe spectroscopy to more complex coincidence measurements, without the need for additional hardware.

Another readout system capable of achieving great temporal (and consequently spatial) resolutions in conjunction with CDL detectors is the time-to-amplitude converter (TAC) [28,29]. While a TDC quickly (in the order of tens of nanoseconds) translates time occurrences into digital timestamps, TACs convert time intervals into voltage levels that must later be converted to digital numbers using analog-to-digital converters. This factor severely restricts the acquisition rate to no more than a few hundred kCounts/s and imposes a minimum event-to-event time, or dead-time, that is currently too high for many applications.

Demands for multi-hit acquisition have driven researchers to seek out TDCs as an alternative to TACs in CDL systems. This decision significantly increases the count rate capabilities, from hundreds of kCounts/s to several MCounts/s and lowers the dead-time from hundreds to tens of nanoseconds. Moreover, although recent examples of TACs implemented within an FPGA can be found in the literature [30], TDCs offer a more researched branch of time-interval meters on FPGA, allowing for a simpler and more stable solution.

Generally, in all systems described in the literature, it is not possible to modify the electronic characteristics (such as the number of channels, resolution, dead time, and count rate) to tailor the acquisition system to a specific request. For example, it is not possible to increase the count rate by lowering the resolution or vice versa without corresponding hardware changes. In this work, however, we have presented an 8-channel architecture that is fully adjustable and features cutting-edge performance. Both the data processing system and the TDC are built on separate FPGAs with firmware that can be fully adapted to experimental needs. Table 3, where the first column lists the introduced detection system’s performances, provides an overview of these solutions.

## 6. Conclusions

In this paper, we present a dual-FPGA reconfigurable electronics system for time-resolved detectors, capable of adapting to different measurement scenarios. The proposed model is based on a state-of-the-art TDL-TDC and high-speed modules for coincidence measurements, both implemented on an FPGA.

The strong modularity of the developed instrument allows tasks to be separated on multiple highly specialized boards, so that one FPGA is focused solely on T/D conversion and a second FPGA is entirely dedicated to the more canonical role of FPGA, i.e., high-speed data processing and transmission and coordination of all signals from one or more sensors involved in the measurements. This architecture allows the code related to the two functions to be modified as needed, without the modifications introduced for one function degrading the performance of the other. A transceiver-based interface between the two FPGAs is implemented to sensibly reduce the crosstalk and obtain an overall temporal precision below 12 ps r.m.s.

We have demonstrated through various on-bench and field measurements that the results of this approach are state-of-the-art, and we have specifically sought to separately show the contributions in terms of temporal and spatial resolution brought by the various sections that make up an entire cross-delay anode-based detector, a system that more than any other can benefit from these electronics.

## Figures and Tables

**Table 1:** Logic resources used by the Cyclone^®^ 10 and Artix^®^ 7.

Role	Logic Utilization	FF	BRAM	DSP
Intel Cyclone^®^ 10	5750/38,000	11,890/152,000	74/382	0/125
(FPGA-Master)	[ALMs]		[M20K]	
AMD Artix^®^ 7	41,025/134,600	58,845/269,200	49.5/365	29/740
(FPGA-TDC)	[LUTs]		[36K]	

**Table 2:** Performance of the proposed FPGA-based TDC.

Feature	Value
Number of Channels	Reconfigurable up to 9
Maximum Dead Time	7 ns
Maximum Channel Rate	200 MCount/s per Channel
Channel Resolution (LSB)	2.34 ps
Channel Precision	<12 ps r.m.s.
FSR	0.145 ms
INL	<4 ps over a range of 500 ns

**Table 3:** Summary of the state of the art.

Feature	This Work	[25,26]	[27]	[28,29]
Spatial resolution [μm FWHM]	31.54	20	7	-
Time precision [ps r.m.s.]	<12	<100	<200	5
System type	TDC	Cross Strip	Pixel	TAC
Detector count rate [Count/s]	10 M	5 M	>200 M	-
Readout dead-time [ns]	7	80	-	150
Reconfigurability	YES	NO	NO	NO

## Data Availability

The raw data supporting the conclusions of this article will be made available by the authors on request.
